# Consent Requirements for Testing Health Policies: An Intercontinental Comparison of Expert Opinions

**DOI:** 10.1177/15562646221076764

**Published:** 2022-02-10

**Authors:** Astrid Berner-Rodoreda, Shannon McMahon, Nir Eyal, Puspita Hossain, Atonu Rabbani, Mrittika Barua, Malabika Sarker, Emmy Metta, Elia Mmbaga, Melkizedeck Leshabari, Daniel Wikler, Till Bärnighausen

**Affiliations:** 1 Heidelberg Institute of Global Health, 9144 Heidelberg University, Germany; 2 Social and Behavioral Interventions, Johns Hopkins Bloomberg School of Public Health, Baltimore, USA; 3 Department of Health, Behavior, Society and Policy, 242612 Rutgers University, Piscataway, USA; 4 Center for Population-Level Bioethics, New Brunswick, USA; 5 3710 McMaster University, Hamilton, Canada; 6 BRAC James P Grant School of Public Health, 246826 BRAC University, Dhaka, Bangladesh; 7 Department of Economics, 95324 University of Dhaka, Dhaka, Bangladesh; 8 6029 Radboud Universiteit, Nijmegen, Netherlands; 9 School of Public Health and Social Sciences, 92976 Muhimbili University of Health and Allied Sciences, Dar es Salaam, Tanzania; 10 1857 Harvard T.H. Chan School of Public Health, Department of Global Health and Population, Boston, USA

**Keywords:** in-depth interviews, informed consent, research ethics, developing countries, international research, public health research, risks, benefits, and burdens of research/ beneficence and non-maleficence, bioethics

## Abstract

Individual informed consent is a central requirement for clinical research on human subjects, yet whether and how consent requirements should apply to health policy experiments (HPEs) remains unclear. HPEs test and evaluate public health policies prior to implementation. We interviewed 58 health experts in Tanzania, Bangladesh and Germany on informed consent requirements for HPEs. Health experts across all countries favored a strong evidence base, prior information to the affected populations, and individual consent for ‘risky’ HPEs. Differences pertained to individual risk perception, how and when consent by group representatives should be obtained and whether HPEs could be treated as health policies. The study adds to representative consent options for HPEs, yet shows that more research is needed in this field – particularly in the present Covid-19 pandemic which has highlighted the need for HPEs nationally and globally.

## Introduction

Informed consent is a central requirement of clinical research on human subjects (Beauchamp & Childress, 2001; Bromwich & Millum, 2015; CIOMS 2016; Dworkin, 1988; Faden & Beauchamp, 1986; Manson & O'Neill, 2007; Miller & Wertheimer, 2010; National Commission for the Protection of Human Subjects of Biomedical & Behavioral Research, 1979; Schaber & Müller, 2018; U.S. Government, 1949; World Medical Association, 1964) yet it is unclear to what extent that requirement should apply to the testing of public health policies.

The need for basing public health policies on scientific evidence has been raised for many years (Petticrew et al., 2005; Wanless, 2004) with Petticrew and colleagues urging action. “We cannot continue to lament the state of the public health evidence base while overlooking the many opportunities to improve it” (Petticrew et al., 2005:756). Yet health policy experiments (HPEs) which test different policy interventions and evaluate them before deciding on and implementing a particular policy were already conducted in the 1970s and 1980s (Manning et al., 1987). HPEs that test different financial incentives or health insurance models for end users have generated evidence for health policies in the Global North (e.g., the Rand and Oregon experiments in the USA) as well as in the Global South (e.g. the universal health insurance program in Mexico and the free healthcare program in Ghana) (King et al., 2009; Newhouse & Normand, 2017; Powell-Jackson et al., 2014). HPEs involve large population groups or clusters and “resemble trials of treatment or diagnostic strategies more than trials of drugs or devices” (Newhouse & Normand, 2017:2166) thus being considered less risky than clinical trials that test new drugs, vaccines, devices, medical procedures and prevention approaches of unknown efficacy (https://www.who.int/topics/clinical_trials/en/). Accordingly, some argue that informed consent requirements for clinical trials cannot be simply applied to health policy research, as governments can authorize health policy research within the scope of their work (MacKay & Chakrabarti, 2019), and individuals are not well placed to decide on health policies:

We argue that investigators need not secure participants’ informed consent when conducting government policy experiments if: (i) the government institution conducting or authorizing the experiment possesses a right to rule over the spheres of policy targeted by the research; and (ii) data collection does not involve the violation of participants’ autonomy rights (MacKay & Chakrabarti, 2019:188).

A … limitation of informed consent procedures in medicine is that they are useless for selecting public health policies. Public policies, including public health policies, have to be uniform for populations. We cannot adjust water purity levels or food safety requirements to individual choice, or seek informed consent for health and safety legislation or quarantine restrictions (O’Neill, 2003:4).

Conducting HPEs in cluster randomized trials poses challenges regarding individual consent. This is particularly the case in so-called “cluster-cluster trials” where “the intervention is targeted at a whole group” (Edwards et al., 1999:1407), i.e. the individual is part of the group and can therefore not simply opt out of the proposed intervention. The testing of fluoridated water in selected cities before introducing it as a health policy is an example for a cluster-cluster trial. The individual in these cities would not have the option to leave the study, and could only withdraw from it by relying on purchased mineral water instead. By contrast, individual-cluster trials offer individuals in the group “autonomy” (Edwards et al., 1999) in their decision to participate. Trialing population-based screening for a specific disease by randomizing health facilities is an example for an individual-cluster trial. In the facilities where the screening is offered, the individual would still be able to opt in or out.

While some scholars argue that in trials where individual informed consent is difficult to attain, the research team could apply for a waiver (Anderson et al., 2015; Taljaard et al., 2013, 2017), others contend that consent should be sought from political representatives, often at the local level, or from representatives of affected communities including community advisory boards (Kilama, 2010) or guardians (Hutton, 2001).

In this article we characterize health experts’ positions from three different countries and continents, a low-income country in Africa, a middle-income country in Asia and a high-income country in Europe on consent requirements in HPEs. We described HPEs to interview partners as “testing the impact of alternative models of health care and other policies that shape population health”, i.e. a scientific approach, often in the form of a randomized control trial, for selecting a proven health policy rather than simply choosing and implementing a health policy among various options. Through this study we aimed to discern whether interview partners thought that HPEs require the special consent of study subjects as most clinical trials do or, alternatively, they do not require any consent beyond the background approval of a legitimate or democratic national political body and authorized experts of the health policy that they test. We also explored whether HPEs call for other forms of consent.

Our study adds to the scarce literature on the ethics of informed consent in health policy experiments with regard to low and middle-income countries (Pratt et al., 2017).

## Methods

### Study Settings

This study was conducted in Tanzania, Bangladesh and Germany – three countries with differing economies, socio-political and cultural contexts and geographic locations. This allowed us to explore commonalities and differences in the perception of informed consent requirements across diverse settings, and it allowed our team to build on longstanding research collaborations. For study country details, see Table 1.

**Table 1.** Social and Economic indicators**^1^**.

General Information and Social and Economic Indicators	Tanzania	Bangladesh	Germany
Population in million	58.1	166.4	82.3
Population Growth Rate (%)	3.1	1.2	0.2
Urban Population (% of total pop.)	33.8	36.6	77.3
Life Expectancy at Birth (male/female) in years	64.8 / 60.8	72.9 / 69.8	82.9 / 77.9
Economics: Gross domestic product (GDP) in million US$	47 653	220 837	3 477 796
World bank classification^2^	Low-income country	Lower middle-income country	High-income country
Health: Current expenditure (% of GDP)	6.1	2.6	11.2
Physicians (per 1 000 pop.)	∼0.0	0.5	4.2
Political system	A unitary presidential multi-party republic with executive powers predominantly lie with the President**^3^**	A parliamentary democratic republic in which executive powers lie with the Prime Minister^4^	A parliamentary democratic republic in which executive powers lie with the Prime Minister^5^
Education: Government Expenditure (% of GDP)	3.5	2.5	4.9
Primary gross enrollment ratio (f/m per 100 pop.)	82.0 / 79.5	122.1 / 115.2	102.1 / 102.7
Tertiary gross enrollment ratio (f/m per 100 pop.)	2.7 / 5.2	14.2 / 20.3	65.6 / 66.9

^1^ UN data from 2018, see http://data.un.org/en/iso/de.html.

^2^ https://datahelpdesk.worldbank.org/knowledgebase/articles/906519-world-bank-country-and-lending-groups.

^3^ http://www.tanzania.go.tz/home/pages/1.

^4^ http://thecommonwealth.org/our-member-countries/bangladesh/constitution-politics.

^5^ https://www.bundestag.de/en/parliament/history/parliamentarism/frg_parliamentarism/frg_parliamentarism-200324.

### Study Design

This qualitative research is based on country case-studies (Merriam, 1998) in the form of face-to-face in-depth interviews with health experts. National interviewers conducted the interviews in the national language or English. The international research team held weekly debriefings to learn from each other's experience, inform the interviewing process and to ensure conformity across the regions. This qualitative study forms part of a study to develop a global ethics framework for health policy experiments which will be informed by both – normative considerations as well as the views and opinions of health experts. This paper reports empirical research that contributes to a collaborative effort to design an ethics oversight mechanism for health policy experiments.

### Sampling and Data Collection

Interview partners (IPs) were purposively selected from the following five sub-groups of health experts: researchers, medical doctors, policy-makers, representatives of non-governmental organizations (NGOs) and ethicists or members of ethics commissions by using snow-ball sampling (Patton, 1990).

We piloted the interview guide with health experts from November 2018 to January 2019 (Tanzania n  =  6, Bangladesh n = 7, Germany n  =  10). The full study team, composed of researchers from each country team, met in Tanzania in January 2019 to discuss the pilot findings and to further adapt the interview guide. As set out in the Introduction, we included a definition of HPEs in the interview guide to mitigate definitional concerns that emerged in piloting. The interview guide consisted of concrete examples of a health policy (a government raising the number of health staff in order to improve quality care in the country), a health policy experiment (the testing of national syphilis screening by randomizing health facilities) and a clinical trial (testing a new medication) (Berner-Rodoreda et al., 2021). IPs were asked to provide their opinion on the respective consent requirements, which were then probed further. Other HPE examples utilized in interviews consisted of testing fluoridation of water in cities before introducing it nationally or testing genetically modified mosquitoes to combat zika and dengue in a confined area before releasing them in a wider infested area.

### Analysis

The research team audio-recorded all interviews and summarized their contents in debriefing notes, which were shared and discussed in weekly skype calls (McMahon & Winch, 2018). Debriefings also served to find commonalities and differences across countries. A more detailed description of findings in Germany in relation to interviewing techniques has been published elsewhere (Berner-Rodoreda et al., 2021). Research assistants and team members transcribed the interviews. All interviews were quality checked and coded in Nvivo (Tanzania and Germany) or Atlas.ti (Bangladesh). The team developed a codebook that was initially based on the interview guide and debriefing notes, but gradually included inductive codes which emerged from the data (Charmaz, 2017; Creswell, 2003). The codebook was further adapted during weekly debriefing sessions, and transcripts were re-coded. One interview was coded by all three country teams and compared. The international team specified codes further when coding varied between countries, and discussed and compared findings in weekly skype calls.

In this analysis we considered IPs’ understanding of informed consent regarding HPEs in order to compare commonalities and differences across the three countries.

### Ethics

This study was approved by the Heidelberg Ethics Commission in Germany (S-291/2018) (Berner-Rodoreda et al., 2021), the Institutional Review Board of BRAC James P. Grant School of Public Health, BRAC University in Bangladesh (2018-018-IR) and the Muhimbili University of Health and Allied Sciences Institutional Review Board (DA. 282/298/01.0C) in Tanzania.

All interview partners received oral and written information about the research project and provided informed written consent prior to being interviewed. Interview transcripts were given pseudonyms in order to protect the identity of the interview partner (Berner-Rodoreda et al., 2021).

## Results

### Interview Partner Characteristics

The international team conducted interviews (n = 58) between February 2019 and September 2019; the number of interviews varied according to data saturation: Tanzania (n  =  18), Bangladesh (n  =  20) and Germany (n  =  20), see Table 2. Further details on the German sample have been published elsewhere (Berner-Rodoreda et al., 2021). Interviews across the three countries lasted on average 60 min. All country teams experienced difficulties scheduling interviews with policymakers; Bangladesh also encountered challenges in identifying ethicists.

**Table 2.** Characteristics of Interview Partners.

	Tanzania	Bangladesh	Germany	Total
**Number of interviews**	**Total**	**Total**	**Total**	
	**18**	**20**	**20**	**58**
**Gender**				
Male	**9**	**15**	**10**	**34**
Female	**9**	**5**	**10**	**24**
**Age**				
up to 30	**3**	**1**	**2**	**6**
31-40	**7**	**7**	**5**	**19**
41-50	**6**	**8**	**3**	**17**
51-60	**0**	**1**	**5**	**6**
61 +	**2**	**3**	**5**	**10**
**Education**				
up to Bachelor's Degree	**2**	**3**	**3**	**8**
up to Master's Degree	**9**	**4**	**4**	**17**
up to PhD	**7**	**13**	**13**	**33**
**Health Experts**				
Medical	**4**	**8**	**4**	**16**
Policymakers	**2**	**1**	**2**	**5**
**NGO representatives**	**4**	**4**	**4**	**12**
Ethicists	**4**	**1**	**4**	**9**
Researchers	**4**	**6**	**6**	**16**

In Tanzania and Germany gender parity of IPs was reached, in Bangladesh 3/4 of IPs were male. A majority of IPs were above 40 years in Bangladesh and Germany and below 40 years in Tanzania. In Germany, older IPs tended to be male and hold a doctorate (Berner-Rodoreda et al., 2021); in Bangladesh, all women and in Tanzania more women than men held doctorates. Across all countries, a majority of policy-makers had a medical background. The youngest IPs were 26 years old (Tanzania and Germany), the oldest was 75 years old (Germany).

### Interview Partners’ Views on Health Policy Experiments and Informed Consent

#### Commonalities Across the Three Countries

There were four substantial points of agreement among interview partners (IPs) across the three countries. First, prior to an HPE, experts should provide a scientific evidence-base or expert opinion on the benefits of such an experiment outweighing potential risks. Second, no prior informed consent is needed in specific situations such as: emergency situations; when testing routine procedures; or when undertaking studies where an individual can opt-out. Third, affected populations should receive information about intended HPEs prior to their introduction. Some IPs across the three countries regarded prior information for some study designs as more important than prior informed consent. Finally, interventions regarded at higher risk for individuals need individual consent or engagement such as a referendum or community dialogue.

Due to unfamiliarity with the term and concept of “health policy experiments” (as testing of health policies is not yet a standard procedure in countries irrespective of their geographic location or income situation), we noticed IPs sometimes switching between “health policy experiments” and “health policy” when answering questions. IPs also tended to use “consent” and “consultation” interchangeably particularly with regard to community or stakeholder consent. We will further elaborate these points.

##### Experts Should Weigh in

Across countries, IPs felt that experts should be consulted before conducting an HPE to ensure that it is a decision based on scientific evidence and local knowledge, as illustrated by the following quotes:

The gold standard would be to get all experts to the table, let them thrash it out and exchange all the arguments. (…) I always try to ensure that people with controversial opinions are among them…If I have planned this right and the experts agree then I can say, this was the expert opinion and we will stick to this and this is the result. (policy-maker, Germany)

Those who deal with diseases should be consulted prior to the HPE. The HPE should be implemented after they agree, considering the statistics, the pathophysiology of the disease. Otherwise resources will be wasted. (medical doctor, Bangladesh)

The one to decide is not from the Ministry [who] just sits in his office and says these are [the] places. When we need to choose facilities, it should be participatory, you need to involve the people who are dealing with those problems like the medical personnel from the particular district or particular villages. (researcher, Tanzania)

Despite general agreement, the statements display regional nuances. For the German IP, involving experts with different and even opposing opinions was an important factor as some IPs also viewed experts critically. The heterogeneity of experts would ensure that all aspects are considered. The Bangladeshi IP underscored medical expertise, and the Tanzanian IP highlighted the grounding of the medical experts in the community or district, thus ensuring familiarity with the local context.

For cluster-cluster studies where the individual cannot easily opt out such as in testing the fluoridation of water or releasing genetically modified mosquitoes in a location, some Bangladeshi and German IPs felt that consent by experts played a crucial role.

Interviewer (I): If we imagine that we will randomize cities [for the fluoridation experiment] and we would say, Stuttgart is in the active arm of this study, who would you ask for consent?

Interview Partner (IP): Those that are affected.

I: Who would not be affected?

IP: Yes, sure.… Well, in this case you would have to add this to the drinking water generally. Then it will become difficult.

I: At what level would you say this decision should be made?

IP: At the level of experts in any case.

I: You mentioned before that the affected should also consent?

IP: Yes, I got into a conflict. In the case of fluoridating water, you cannot say, “would you like to take part or not?” If I live in this city, then there’s no alternative where I could give consent. Then this can only be done by the experts. (medical doctor, Germany)

Perceiving a need to involve experts prior to testing an HPE did not imply consensus on the composition of expert groups or research ethics committees which some IPs across all three countries felt should be consulted prior to the intervention; variances will be discussed in the section on differences below.

##### No Prior Consent is Needed in Specific Situations

If the experiment was linked to improving routine procedures in a medical facility such as introducing electronic data systems in a hospital or if it is related to an emergency such as an outbreak of a contagious disease, where governments needed to act quickly, IPs felt that asking for consent would either not be needed or may make an intervention less effective.

I: Can you think of any situation when the government or the research team may not need to take any consent?

IP: I say only in case of public health emergencies. (NGO representative, Bangladesh)

IPs also felt that the general population did not need to be asked for consent before the introduction of an HPE, if they could decline an intervention during implementation, i.e. opt out of an intervention or decide not to opt in as exemplified by this quote:

If a person is informed and can opt out before taking part in the experiment, then s/he is giving consent. (medical doctor, Bangladesh)

##### Affected Populations Should be Informed About HPEs

Across all three countries and sub-groups of health experts, IPs felt that prior awareness and information about an experiment was an obligation toward the affected population irrespective of what form of consent may be used. A German health expert expressed this as follows:

I live near Mannheim and when I drive, I see this industrial plant. Sometimes the smoke is black, sometimes white (…). But if there was green smoke, and I would not know why, I would feel uncomfortable because an experiment takes place and nobody in the vicinity is informed about it. (…) At least people need to be informed. This is the be-all and end-all. This needs to be done. (researcher, Germany)

IPs mentioned newspapers, radio, posters and social media as sources of information which may increase acceptability for an HPE. Some IPs also described prior information as a means to aid individual consent and mitigate the spread of false information.

This [information] can ensure a slightly more enabling environment for the experiment. You know other vital services like immunization can be affected negatively because of rumors and gossips [when information is not provided]. That is why broad awareness is important. Then it should move towards individual consent which is more private and confidential for the experiment. (researcher, Bangladesh)

Utilitarian considerations or information that this may benefit the “common good” was also mentioned by a number of IPs across the three countries as possibly facilitating participation in HPEs.

##### Risky Interventions Need Individual Consent or Engagement

Across all three countries, IPs felt that individuals should be asked at least in the form of a community dialogue or survey, if not a referendum or some other form of individual consent, if they perceived the HPE to involve personal risks. High risk perceptions by individual IPs were linked to cluster-cluster studies, which allowed the individual no opt-out and for which risks could not be ruled out. The testing of fluoridated water in different cities to improve dental health or the release of genetically modified mosquitoes to reduce Zika or Dengue (Langston, 2016) served as examples.

Okay, this is a tricky one [the release of genetically modified mosquitoes], but I think it depends on the population and how exposed they are, which area you are researching on, but in all cases, I think it is good that you involve them because at the end of the day whatever you do may have direct or indirect impact on them. (medical doctor, Tanzania)

With regard to the same example, one German ethicist expressed skepticism about a utilitarian approach when the measures might potentially harm an individual.

The inclination towards a referendum was often based on IPs’ general attitudes or preferences, such as relying on tap water and being opposed to adding substances to drinking water or insufficient information about the potential benefits and dangers of genetically modified mosquitoes.

IP: That’s where the fun ends. This is of course a decision I want to make.

I: Why do you want to make this decision yourself?

IP: Because water is life-blood, the most precious thing we have on this planet and which I regard in high need of protection. And it would be a higher good to think of the next generation. (NGO representative, Germany)

As I am thinking about the general people, it is important to know whether they really want that water. For example, if the people say that they don’t want it…In that case I think it is important to take consent from the people of that community that something is being added to their water supply. (medical doctor, Bangladesh)

With issues like that I would lean towards a referendum and not go through representatives. I mean, I don’t know what these mosquitoes can do, they can probably bite or whatever but I would like to be part of the discussion and ask questions individually. It’s the same with drinking water. Yes, I think, it would depend on the consequences and what this experiment involves. (researcher, Germany)

The last quote demonstrates the uncertainty of the IP who is leaning towards a referendum, as the IP perceives some risks and would like to have individual questions answered. To ensure that people are well informed about advantages and possible risks, some IPs felt that a community dialogue with specialists or public deliberation could be an appropriate alternative method of involving and discussing the experiment with individuals.

Why should you fluoridate? That’s what people would want to know. Is this good for me? What are the dangers? What positive effects does it have? And then people understand. A community dialogue, if it is carried out successfully, is the ideal way. (ethicist, Germany)

In this case, community engagement is much more important than consent. Ownership of the community (regarding) why are we doing this. Public deliberation is very important. And if there is substantive opposition, you do not do that experiment. (researcher, Bangladesh)

Individual risk perceptions coupled with information needs on a particular health policy experiment thus played a major role for IPs in their perception of the need for individual consent versus other consent forms.

##### Shifting Between “Health Policy Experiments” and “Health Policies”

Most IPs were not initially familiar with the term “health policy experiment” (HPE) regardless of where they lived or their professional health expertise. This emerged when piloting the first interviews. A definition was therefore incorporated into the interview guide. Yet in the interviews, the answers of IPs frequently switched from discussing an HPE to discussing a health policy and vice versa, and it was not unusual for the interviewer to clarify whether the IP was talking about an HPE or a health policy. To illustrate this general tendency, we provide the example of a German health expert who switched twice to the discussion of a health policy under consideration in Germany at the time (making organ donation an opt-out rather than an opt-in policy) when discussing requirements for an HPE (the testing of national syphilis screening) thus clouding the differences between the two concepts:

In general, I tend to be restrictive in considerations of an opt-out. If I can use a different example where this is presently being considered… organ donation. I personally tend towards opt-in rather than opt-out. My great misgivings would be that I would potentially have to justify myself when I say, I don’t want this test [syphilis screening] …

I think additional information is important for my personal decision-making but if it is too much it will lead to resistance. Let me take the example of organ transplants again. (…) This problem [organ donation] is vehemently imposed on me through TV spots, opinion pieces, where pressure is mounted on me. This leads to me saying: “No, I would like to decide this privately.” It depends on the aggressiveness of such a campaign, such an information campaign. (researcher, Germany)

##### Using “Consent” and “Consultation” Synonymously

We also found that IPs across all countries often used the terms “consent” and “consultation” interchangeably. Both terms referred to the need of soliciting the willingness of the group or individuals to be engaged in research or experiments as expressed by this Tanzanian ethicist:

Since this is research, consultation should be to both the health facilities and the individuals to be tested. The health facilities need to approve their engagement in providing the diagnostic and treatment services and the individuals need to agree or disagree to doing the test. (ethicist, Tanzania)

#### Differences Between the Three Countries

Table 3 shows differences in health experts’ perception of informed consent requirements for HPE across the three countries. While not all IPs in each country held the same opinion, we compare and contrast the overall perception of IPs between the three countries. The sub-sections that follow elaborate each row in the table.

**Table 3.** Country Differences on IPs’ Perspectives on Informed Consent Regarding Health Policy Experiments.

Tanzania	Bangladesh	Germany
**Should everybody consent?**		
Most IPs preferred individual consent (e.g. a referendum).	IPs were divided on this question.	Many IPs problematized a referendum, yet exposure to high personal risk would warrant individual consent or at least a community dialogue or survey.
**Should group representatives consent?**		
Most IPs mistrusted representative consent and valued it only if based on majority opinion of community; some suggested involvement of community advisory board.	Most IPs preferred representative consent; some suggested institutional consent through health authorities as alternative.	Most IPs preferred representative consent as the most appropriate consent form in a representative democracy, unless the HPE was perceived as risky.
**How should expert and research ethics committees be composed?**		
IPs proposed committee composed of health experts.	IPs proposed committees composed of all major stakeholders.	IPs proposed committees composed of independent health experts, key stakeholders and ethicists; one IP included philosophers and older/wiser people.
**Should HPE be subject to the same ethics requirements as Health Policies?**		
Most IPs saw HPEs as requiring consent unlike health policies which IPs viewed as already proven to work efficiently and safely.	Many IPs emphasized the experimental character of HPE and thus needing consent, yet some IPs felt that HPEs could be treated like HPs.	Many IPs emphasized the experimental character of HPE and thus needing at least ethics approval, yet some IPs felt that HPEs could be treated like HPs.
**Should consent be obtained from those implementing HPEs?**		
Most IPs preferred to obtain consent from implementers.	No clear trend – some preferred, others objected to obtaining consent from implementers.	Many IPs preferred to obtain consent from implementers with some changing their mind during the interview.

##### Should Everybody Consent?

Among the three countries, Tanzanian IPs advocated the strongest for individual informed consent in relation to HPEs. By contrast, in Bangladesh, while many IPs felt that individual consent was needed, the only ethicist interviewed held the opposite opinion, and some IPs perceived individual consent generally as problematic in cluster trials. In Germany, personal risk perceptions guided IPs’ views on consent requirements, and many IPs considered holding a referendum prior to an HPE unnecessary in a representative democracy. Three country quotes illustrate different perceptions:

What is necessary is that before implementation of anything in the community, we need the group to be aware and to consent to the new intervention. And this should be the approach to anything, any research, any intervention, any policy (NGO representative, Tanzania).

But the question is, who will represent that community? Are you again going to conduct a referendum for this? Yes/no vote? And the person you are going to choose from that community to vote, will he actually represent the community? So that’s a problem. So, these community trials are ethically cumbersome. (medical doctor, Bangladesh)

IP: We have an extremely strong dominance of a representative democracy. This means that the majority of our ‘referendums’ are through elections. And then I’d say, these are the representatives of the people in democratic structures and committees… I would restrict referendums to extreme situations.

I: How would you define ‘extreme situations’?

IP: Such as questions of national security – but even then, I am not sure, if I would do it. (ethicist, Germany)

While IPs in Tanzania favored individual consent, IPs in Bangladesh and Germany mentioned the Brexit experience as a deterrent against a referendum, as “one would have to consider that many decisions are based on emotions” (policy-maker, Germany). Further reasons among German and Bangladeshi IPs for not asking for individual consent prior to the experiment included: people being unable to make an informed choice on a complex matter for which they may lack the necessary background information; not wanting to base a future scenario on people's past experiences; the self-selection of people who make use of a referendum that may include only people with strong opinions on the matter; logistical difficulties in conducting a referendum; methodological inappropriateness for cluster-trials; seen as unnecessary when a government has the authority to conduct experiments; delaying interventions in case of emergency situations. If the experiment involved the danger of increasing stigma, some IPs in Bangladesh felt that it suffices to ask only affected patients when carrying out the experiment rather than getting prior consent for the experiment from the entire population.

##### Should Group Representatives Consent?

Views on consent by local representatives varied across the three countries. The term ‘representative’ included political representatives such as members of a city council as well as advisory board members or other community representatives. Most IPs in Germany felt that democratically elected representatives at the appropriate level of government could decide on HPEs as long as the benefits of the experiment outweighed the risks – this was particularly the case for randomizing clinics in the syphilis-screening example. In Bangladesh, some IPs believed that local government or leaders can provide consent on behalf of the people and that this may have a positive effect on the community, yet one could also have institutional consent in form of a health authority or important health institution. In Tanzania, IPs favored joint consent of the local population with their leaders to prevent undue political influence by political leaders. Table 4 presents illustrative quotes on the positions and arguments in support of and against representative consent as well as in support of requiring joint consent by leaders and the affected population.

**Table 4.** Positions and Specifications on Consent by Local Group Representatives.

In support of local group representatives and committees consenting for affected population	Against local group representatives consenting for affected population	In support of joint consent between local group representatives and affected population
if not dominated by particular personal interests I’m a fan of parliamentary democracy. If the system works and is not too much dominated by personal interests, then I would say, it is fine and no further level needs to be involved…. I could imagine that … one would involve the community, perhaps a council or a council of elders in Africa. That one would discuss this with them and provide reasons why we want to do this here and not over there. I could imagine doing it this way and not to ask every individual for consent. (Germany, medical doctor)	lack of medical expertise The challenge I see is that when it comes to this very purely medical conditions there is much asymmetry of information between the non-health professionals to health professionals. (Tanzania, policy-maker) …at communal level - no. They (city counsellors) can be consulted but they can’t gauge the interests behind this. (Germany, ethicist) (Berner-Rodoreda et al., 2021)	involving local structures and local population Even if there are no existing guidelines on how to take group consent, there are structures in the community example the health committees that represent people of different cadres in the community so these committees should be consulted to provide the community consent but still individual consent is mandatory because those who are going to get the test are the individuals. (Tanzania, ethicist) Then there must be an organ that connects the research team or the government to the community, an advisory board and this is very common in vaccine trials. This board has all the expertise and it will help to sensitize the community and obtain the community consent before seeking the individual consent at the health facilities where the testing will be done (Tanzania, ethicist)
if benefits outweigh risks I guess the parliament or city council of that county that has seen the problem said: there is no other solution to combat these mosquitoes. Then we will do it. That is also a democratic decision. If they tested and found out that the safety of these modified insects is greater than the damage that these insects can cause, then I’d have no problem with the county making this decision… Yes, I think a parliament should be able to make this decision… They (democratic institutions) have to decide, if a certain project should be conducted. (Germany, medical doctor)	local representatives might have vested interest But what do you do, if you have a regional CSU (Bavarian conservative party) councilor in Bavaria who is a board member of GSK (pharma company Glaxo-Smith-Kline)? Are they the right ones? I don’t think so. (Germany, ethicist) (Berner-Rodoreda et al., 2021) We often think that the opinions of local community leaders are everyone's opinions. That sometimes may not be necessarily true. The ones whom we are calling influential, they are basically politically influential. They have vested interest which most of the time reflects their own agendas rather than something that supports the population or the community. That is why it is misleading to consider their consent as community's consent (Bangladesh, researcher)	local leaders to represent after consultation with local population . leaders should not make their own decision without consulting the people they are representing. They will have to go to the people they are leading and tell them that we have been asked to take part in an experiment on this and that and get their views, so based on the community feeling the local leaders can make decisions on whether to consent on their behalf or not.” (Tanzania, researcher)
local population trusts in judgement of local representatives If the local government is involved, more community people will be influenced to do the test… If an outsider comes in and asks the community to take part in the HPE, they may not want to do it. But if the people whom they know, the local government tells them to take part in it, then they are more likely to do it. (Bangladesh, medical doctor) In a rural area, a chairman of Union *Parishad* is an influential person. So, his/her consent is important. There are health committees in each Union *Parishad* which comprise of Union *Parishad* members. It might be necessary to discuss with that committee and may require group consent, if not individual. It might become more difficult in the urban areas as such gatekeepers are absent.” (Bangladesh, researcher)	inappropriate level of decision-making The local leaders are not involved in formulation of any kind of health policy. After the research (HPE), the local government will not contribute in any way in the health policy changes. It is done centrally by the researchers. The researchers discuss with the governing and implementation bodies like ministry of health and directorate of health services, and these bodies formulate the health policies. Local government does not play any role, so it is not necessary to take their consent (Bangladesh, medical doctor)	
group reps should consent, not individual reps I would not ask individual councilors in Indonesia – no way. In Stuttgart I first thought we could ask a zone mayor because I wondered who else should be involved, what lower political level should be included? But I would say, it should be the local councilors. (Germany, NGO representative)	Alternative form of ‘institutional consent’ We can take informed consent from institutions instead of an individual. Say I have some patients admitted here, I’ll collect their data – not interrupting their treatment. We can take institution's consent for that. (Bangladesh, medical doctor)	

While mainly Bangladeshi and German IPs presented arguments for consent by group representatives, some IPs also expressed reservations towards representatives who were believed to lack medical knowledge or to have vested interests. In order to improve “informed” consent by local leaders or representatives, Tanzanian IPs suggested thoroughly informing leaders before involving them in the decision:

… if we engage them, you will start first by giving them knowledge on why this should be done and once we are at the same level of understanding why syphilis should be tested in this community then you can involve them to make decisions otherwise your decision will fail because of this asymmetry and knowledge and understanding of the community. (policy-maker, Tanzania)

Ensuring that health personnel work in collaboration with local leaders was cited as another option:

… it would make more sense for the health care workers who have more knowledge on syphilis and its consequences to work hand in hand with the local leaders in delivering the message to the communities. This would be an opportunity for health care workers to answer the concerns from the people, thus ensuring the right message is delivered. (ethicist, Tanzania)

Yet, Tanzanian IPs advocated for any consent by group representatives to be based on the will of the people rather than on representatives deciding for them.

##### How Should Expert and Research Ethics Committees be Composed?

While a number of IPs across all three countries suggested prior consultation with research ethics committees or independent committees composed of experts, ideas about the preferred composition of these committees varied: a Tanzanian ethicist suggested consulting different health stakeholders; a Bangladeshi researcher proposed including all major stakeholders such as government, civil society organizations, various health experts and researchers; and in Germany ideas varied: some felt the committee should include health or technical experts, some added key stakeholders (insurance companies, expert associations, representatives of medical doctors, representatives of policy-makers, patient representatives). A German ethicist recommended including ethicists and philosophers - “people who have a broad overview of different aspects” - for such a committee, which could be used for both health policies and HPEs. This ethicist was in favor of having older people serve on a committee as they have “stored up much wisdom” (ethicist, Germany) and spoke of a “polylog of the wise”. The independence of such committees and the incorruptibility of people serving on them was the primary concern of two ethicists. “If people have serious conflicts of interests, they should not be allowed to be part of the committee or at least not allowed to vote in the committee.” (ethicist, Germany). Many IPs did not provide details of the composition of these committees or who they defined as experts.

Some IPs felt that expert advice would be sufficient to conduct an HPE, others viewed clearance by a research ethics committee as a precondition. In Tanzania, experts were seen as aiding the government; their opinion was not seen as replacing consent by the local population, and very few mentioned the involvement of research ethics committees.

##### Should HPEs be Subject to the Same Ethics Requirements as Health Policies?

In Tanzania, IPs made a clear distinction between health policies and health policy experiments in terms of consent, as expressed by this ethicist.

If the government is implementing a policy and not policy research then there is no need of asking for consent rather they need to inform people of the new policy, why a new policy, what is expected of them and the likely benefits. But if it is policy research then all procedures for research should be followed, it should be reviewed by the ethical review boards to get clearance for its implementation and as I said obtain consent from the community and the individuals involved. (ethicist, Tanzania)

In Bangladesh and Germany, the sentiment that HPEs were still in an experimental phase was largely echoed, yet some IPs believed that HPEs which posed no or little risk to the population or ensured equipoise in the experimental design could be treated the same as health policies with no consent requirement other than a political decision-making process:

If it is an important health issue which you want to test nationwide, I don’t think government needs to take consent from anyone. If they think that it is needed for the benefit of the community, then they can do it. (medical doctor, Bangladesh)

So if a government can say: we are introducing this Health Policy, and we do not need to ask anyone for permission, and everyone needs to do it this way from that particular day onwards, then I do not see the necessity for consent, if they say, we want to test this in a randomized way in some of the clinics beforehand, and if it proves useful, we will conduct it everywhere; if not, then it will not be introduced. But this is also an ethical question. It must not be evident that one way of doing this is clearly superior to the other. (researcher, Germany,)

##### Should Consent be Obtained from Those Implementing HPEs?

While most Tanzanian IPs favored consent from individuals or health facilities tasked to implement a health policy experiment, the picture in Bangladesh and Germany was more diverse. The opinion that consent should be sought from implementers before the HPE to improve cooperation, adapt the approach to the local situation and practice a participatory approach was mainly voiced by researchers and ethicists across the three countries as these three ethicist quotes show:

The government should consult like the medical officers in the districts/regions and the participating facilities to help in deciding whether the suggested approach is feasible or not. (ethicist, Tanzania)

In case of policy experiments, the matter is different. By consent I mean the government can discuss and ask for suggestion. They [the government] can say “look, this policy was implemented elsewhere and it is successful. Can we do something like that?”…[…]… they should come to consensus and should include local implementers because they are the ones who are aware of the problems that arise at the ground level [where the implementation will take place]”. (ethicist, Bangladesh)

IP: Firstly I would naturally make this public in the Republic of Germany or in the relevant country. I would inform all included facilities in detail about the study and I would ask for their consent. (…)

I: Would you view this as prior consent or a prior consultation?

IP: Consent. (ethicist, Germany)

Yet, other researchers in Bangladesh and Germany held the opinion that health personnel should be informed and consulted but not asked for consent about taking part in the HPE, as this would “reduce the generalizability of results” (researcher, Germany) (Berner-Rodoreda et al., 2021).

The implementers at the institutional level, for example DG Health, would be aware of it [the experiment] obviously since they would be implementing. So, there is no question of their consent. At the field level, the ones who would be offering the [syphilis] test to the people should be informed and made aware. Their informed consent is not needed. (researcher, Bangladesh)

We therefore see some commonalities but also varied perceptions across the three countries.

## Discussion

Our multi-country findings show some commonalities in health experts’ opinions such as the need for expert advice and information about the HPE to those affected by it, an agreement that the benefits inherent to the HPE should outweigh risks, no prior individual consent in public health emergency situations or testing routine procedures, yet the need for consent – either individual or community-based – for risky interventions. While IPs across the three countries tended to switch between “health policy” and “HPE” in their answers, they mostly tended to apply ethics criteria for clinical trials to HPEs, with many IPs upholding ethics clearance of the experiment and consent either by individuals or group representatives.

Our study also revealed differences across the three countries: while IPs in Tanzania favored individual informed consent, many IPs in Bangladesh and Germany felt that HPEs needed individual informed consent mainly when the population might be exposed to greater personal risks. For other health policy research, Bangladeshi and German IPs mostly favored consent by representatives. We also noted differences between countries in IPs’ opinions on the composition of expert and research ethics committees, consent from implementers, and ethics requirements for HPE – these underpinned important variations in foci and emphases. Yet being based on qualitative research, these differences should not be interpreted as representative of nationality or country of residence.

Ethicists constituted one sub-group of health experts interviewed. They could either be philosophers/ethicists by training or members of a national or local ethics council. There were no marked differences between ethicist views of HPEs and that of other IPs. While a majority of ethicists felt that implementers of experiments would have to provide their consent as well, this view was shared by some researchers and contested by others. German ethicists brought to the dialogue the considerations that expert committees should be independent with no influence by industry or lobbyists and that patient representatives should be included in expert committees. A majority of ethicists also favored involving research ethics committees or mentioned the need for ethics oversight for HPEs; some viewed information on the common good as an incentive for people to participate in HPEs yet this view was not unique to ethicists. Utilitarianism was, however, also regarded critically by a German ethicist if measures intended for the common good could potentially harm individuals. For ethicists as well as for other health experts, opinions regarding consent could vary based on the examples given.

Since implementation and health system studies are often conducted as cluster-cluster studies, IPs’ perceptions of these studies seem particularly pertinent for HPEs. IPs’ opinions on consent requirements for HPEs across all three countries often wavered from one example to the next – such as when discussing national screening programs, the fluoridation of water, or the release of genetically modified mosquitoes and were often based on their own experience or professional background. Some IPs would consider authorization through a research ethics committee for a national screening program as sufficient, but would lean towards consent by local representatives or even individuals for the fluoridation of water or the releasing of mosquitoes thereby demonstrating that personal risk perceptions played a major role in deciding on consent requirements. Despite the impossibility of eliciting individual consent in cluster-cluster studies, some IPs favored this approach or the closest alternative, e.g. a community dialogue, or a combination of representative and individual consent if the HPE was felt to contain individual risks. This casuist approach which judges each HPE on its own merits or risks is an important empirical finding which needs to be considered in developing normative ethics guidance for HPEs.

### Best Practice

Our research findings on informed consent in relation to participation in HPEs – based on the viewpoints of health experts across three continents – provide important considerations for the further development of normative procedures and standards for ethics oversight of these distinctive experiments. HPEs that are compliant with mechanisms that still have to be developed further would constitute best practices. The following suggestions by health experts in Tanzania, Bangladesh and Germany should therefore be understood in this light: they are likely to inform rather than already constitute best practices.

The following important considerations for group representative consent emerged from our study and add to ongoing debates about the role and requirements for gatekeeper/guardian/community leader/representative consent for cluster permission (Edwards et al., 1999; Gallo et al., 2012; Onwujekwe, Shu, & Okonkwo, 1999; Weijer et al., 2011): (i) representatives in group consent processes should not have personal vested interest in the HPEs; (ii) representative groups or committees should be preferred over individual representation to prevent undue influence and mirror the target population's plurality of opinion; (iii) representatives should seek prior consultation with affected populations and expert advice to ensure that decisions reflect population preferences and are based on evidence; (iv) local representative consent should only be chosen if study benefits clearly outweigh risks, and the decision to conduct an HPE can be made at the local level. Some IPs underscored the importance of collective information approaches and dialogue with the affected population when individual informed consent could not be obtained – a requirement which some scholars have called the “information disclosure obligation” (Berg, 2012; Lignou, 2018).

Whether an independent committee or an ethics council may be the most appropriate organ to decide on and monitor HPEs that carry some risks to the population would have to be decided at country level. IPs suggested to include all major stakeholders such as government representatives, civil society organizations, health or technical experts and researchers of differing opinions, patient representatives or representatives of affected populations, philosophers, ethicists, “old and wise people” and to vet committee members for conflicts of interest.

Some Bangladeshi and German health experts felt that policy experiments can be treated like health policies in terms of consent, if (i) a government is acting within its political scope, (ii) the intervention to be tested poses low risk, (iii) the intervention is not known to be superior or inferior to the present intervention. Our findings showed variation in health experts’ views of low risk. These will have to be considered in developing normative guidance on low-risk HPEs which would allow governments to conduct HPEs without lengthy preceding procedures. Governments would, however, also need to take other considerations such as the costs for trialing a health policy into account.

IPs’ perceptions across the three countries that consent from individuals is neither needed for health policy experiments in emergency situations or severe epidemics (due to urgent societal needs and logistical problems) nor for quality improvement studies echoes insights from previous studies and regulations (Calain et al., 2009; CIOMS 2016; Ernst & Fish, 2005; Faden et al., 2013; Finkelstein et al., 2015; Flory, Mushlin, & Goodman, 2016). The present Covid-19 pandemic has put a spotlight on the urgency of conducting HPEs as a means to determine which measures work best for the common good in terms of re-opening schools or (part)-lifting a lock-down to name some of the difficult decisions governments were grappling with (Faherty, Lurie, & Wong, 2020; Fischhoff, 2020; Starr, 2020). Through randomized control trials governments could test, evaluate and identify public health approaches which curb the spread of SARS-CoV-2 rather than continue to “experiment” with health policies on a trial and error basis. Yet there is a dearth of guidance on how to ethically conduct HPEs or general research in severe epidemics (Calain et al., 2009). CIOMS recommends that for studies conducted “under a public health mandate… normally neither ethical review nor a waiver of consent is needed” (CIOMS 2016:39) – the views expressed by IPs in our study that informed consent can be omitted in an epidemic align with these recommendations and with the practice for cluster studies (Flory et al., 2016).

While the present government responses to Covid-19 can at best be seen as unofficial experiments, recent actions and demonstrations in a number of countries have shown that some segments of the population express disregard for various measures which are at present implemented as “emergency measures”. The suggestions by IPs in our study to hold community dialogues where citizens can voice their misgivings and have their concerns addressed by experts could be one promising approach to garner buy-in and support of the affected population and thus improve compliance with measures.

### Educational Implementation

National governments and authorities would have to implement HPEs and would therefore need to be persuaded of their usefulness and would need to decide if an independent committee should oversee HPEs. Such a committee for approving and monitoring HPEs could, if all relevant stakeholders are represented, be a good way of reaching impartial decisions on complex HPEs. The committee's success hinges on all parties being well-informed, all opinions being heard, no stakeholder group dominating proceedings, and a willingness to engage with all known evidence before a decision is made. These conditions, in turn, would require training of all committee members on democratic principles, principles of meaningful engagement, and the scientific and social research evidence base for the HPE. A committee would further require training some members in monitoring skills to ensure that committee meetings are deeply dialogic and are not dominated by a few committee members.

### Research Agenda

The plurality and situation-specific perceptions of informed consent requirements for HPEs by IPs resonate with findings of a recent scoping review (Bachani, Rattani, & Hyder, 2016), which demonstrated that at present no clear ethics requirements exist for HPEs. While requirements for health system studies and HPEs have so far been largely based on consent requirements for clinical research (MacKay & Chakrabarti, 2019), there are no generally accepted guidelines as to whether informed consent is needed or can be waived for implementation or health system studies including HPEs (Bachani et al., 2016; Gopichandran et al., 2016; MacKay & Chakrabarti, 2019). While many authors uphold individual informed consent requirements as a crucial requirement across a range of research trials (Brown et al., 2014; Essack et al., 2010; McRae, Taljaard, & Weijer, 2016; Taljaard et al., 2017), some prefer community consent or consultations with communities (Blom & De Vries, 2011; Weijer & Emanuel, 2000) while others argue for a possibility of no consent in less risky research studies, e.g. pragmatic trials or quality improvement studies (Dal-Ré, Carcas, & Carné, 2017; Taylor et al., 2010). In relation to risk, our study found that risk perceptions are based on contextual and individual characteristics, which merits consideration for future research in terms of developing risk thresholds. Opinions by health experts we interviewed therefore add nuance to the variety of informed consent requirements for HPEs in the literature, especially on consent by representatives, yet call for more empirical research on appropriate consent forms for cluster-cluster trials.

We view the development of ethics standards for HPEs as a staged process. In our opinion normative guidance will have to draw on a variety of sources, including widely-accepted ethics principles as well as considerations involving preferences and perceptions of scientists and of the public thus combining normative considerations with social acceptability and personal perceptions in different countries and locations. Our empirical research has been undertaken to provide data on health experts’ perceptions of consent requirements in diverse social settings. These empirical findings will need to be taken into account in a quest to develop normative guidance for HPEs that shows local relevance across diverse settings.

## Conclusion and Limitations

Our study, based on in-depth interviews with health experts in Tanzania, Bangladesh and Germany, contributes empirical findings to developing a global ethics oversight mechanism for HPEs. The study also adds to the scarce literature on informed consent requirements for health policy research, particularly in low and middle-income countries. Overall, we found more commonalities in the opinions of Bangladeshi and German health experts than between Bangladesh and Tanzanian interview partners. To what extent this is due to a selection bias of interview partners, the socio-political context with both countries being parliamentary democratic republics with executive powers vested in the prime minster, or just coincidence, is difficult to determine. As in all qualitative research, views and opinions by IPs cannot be interpreted to be nationally representative, even if the national context may have a bearing on people's general preferences for one consent form over another.

The fact that health experts across the three countries were not intimately familiar with the concept of HPEs was a limitation of our study, yet a finding in itself as it reflected the current practice of introducing public health policies before testing them. We sought to mitigate this confusion by including a definition of HPEs in the interview guide, but we felt that this proved insufficient in terms of clarifying the distinction between an existing policy and testing or trialing policies. We therefore propose that future empirical research on HPEs include more in-depth briefings on the concept of HPEs before interviews are conducted. We also recommend conducting group discussions to examine if participants can more readily build on each other's arguments in a group situation thereby generating more insights into the complex field of ethics requirements for HPEs.

The ongoing Covid-19 pandemic has forced many public health practitioners, policymakers and lay audiences to consider the value of strategic, coordinated, scientifically-informed endeavors to address (and measure) health interventions at the population level. The need for HPEs for determining the best course of action has hardly ever been greater. We therefore hope that this extraordinary situation will inspire more research into HPEs, their acceptability and ethics considerations for conducting them.

## References

[bibr1-15562646221076764] Anderson M. L. Califf R. M. Sugarman J. ; for the participants in the NIH Health Care Systems Research Collaboratory Cluster Randomized Trial Workshop. (2015). Ethical and regulatory issues of pragmatic cluster randomized trials in contemporary health systems. Clinical Trials: Journal of the Society for Clinical Trials, 12(3), 276–286. 10.1177/1740774515571140. 25733677 PMC4498459

[bibr2-15562646221076764] Bachani A. M. Rattani A. Hyder A. A. (2016). A scoping study on the ethics of health systems research. Developing World Bioethics, 16(3), 124–132. 10.1111/dewb.12117 27038160

[bibr3-15562646221076764] Beauchamp T. L. Childress J. F. , Professor of Philosophy and Senior Research Scholar Tom L. Beauchamp, & Childress J. F. , and University Professor and Hollingsworth Professor of Ethics James F. Childress. (2001). Principles of biomedical ethics. Oxford University Press.

[bibr4-15562646221076764] Berg J. (2012). All for One and One for All: informed consent and public health. Hous. L. Rev., 50(1), 1–40. https://core.ac.uk/download/pdf/214108736.pdf

[bibr5-15562646221076764] Berner-Rodoreda A. Bärnighausen T. Eyal N. Sarker M. Hossain P. Leshabari M. Metta E. Mmbaga E. Wikler D. McMahon S. A. (2021). ‘Thought provoking’, ‘interactive’, and ‘more like a peer talk’: testing the deliberative interview style in Germany. SSM - Qualitative Research in Health, 1, 100007. 10.1016/j.ssmqr.2021.100007 PMC8688150 34977853

[bibr6-15562646221076764] Blom E. De Vries R. (2011). “Towards Local Participation in the Creation of Ethical Research Guidelines.” *Global Summit of National Ethics Committees: An Essential Tool for International Dialogue and Consensus-Building 154* 8(3).

[bibr7-15562646221076764] Bromwich D. Millum J. (2015). Disclosure and consent to medical research participation. Journal of Moral Philosophy, 12(2), 195–219. https://philarchive.org/archive/BRODAC-4

[bibr8-15562646221076764] Brown B. Kinsler J. Folayan M. O. Allen K. Cáceres C. F. (2014). Post-Approval monitoring and oversight of U.S.-initiated human subjects research in resource-constrained countries. Journal of Bioethical Inquiry, 11(2), 119–123. 10.1007/s11673-014-9525-4 24839155

[bibr9-15562646221076764] Calain P. Fiore N. Poncin M. Hurst S. A. (2009). Research ethics and international epidemic response: The case of ebola and marburg hemorrhagic fevers. Public Health Ethics, 2(1), 7–29. 10.1093/phe/phn037

[bibr10-15562646221076764] Charmaz K. (2017). The power of constructivist grounded theory for critical inquiry. Qualitative Inquiry, 23(1), 34–45. 10.1177/1077800416657105

[bibr11-15562646221076764] CIOMS (2016). International ethical guidelines for health-related research involving Humans. Council for International Organizations of Medical Sciences (CIOMS).

[bibr12-15562646221076764] Creswell J. W. (2003). Research design: qualitative, quantitative, and mixed method approaches (2nd ed). Sage Publications.

[bibr13-15562646221076764] Dal-Ré R. Carcas A. J. Carné X. (2017). Who Is willing to participate in Low-risk pragmatic clinical trials without consent? European Journal of Clinical Pharmacology, 73(12), 1557–1563. 10.1007/s00228-017-2332-1 28900674 PMC5684310

[bibr14-15562646221076764] Dworkin G. (1988). The theory and practice of autonomy. Cambridge University Press.

[bibr15-15562646221076764] Edwards S. J. L. Braunholtz D. A. Lilford R. J. Stevens A. J. (1999). Ethical issues in the design and conduct of cluster randomised controlled trials. BMJ, 318(7195), 1407–1409. 10.1136/bmj.318.7195.1407 10334756 PMC1115783

[bibr16-15562646221076764] Ernst A. A. Fish S. (2005). Exception from informed consent: viewpoint of institutional review boards—balancing risks to subjects, community consultation, and future directions. Academic Emergency Medicine, 12(11), 1050–1055. 10.1197/j.aem.2005.06.015 16264073

[bibr17-15562646221076764] Essack Z. Koen J. Barsdorf N. Slack C. Quayle M. Milford C. Lindegger G. Ranchod C. Mukuka R. (2010). Stakeholder perspectives on ethical challenges in Hiv vaccine trials in South Africa. Developing World Bioethics, 10(1), 11–21. 10.1111/j.1471-8847.2009.00254.x 19459900

[bibr18-15562646221076764] Faden R. R. Beauchamp T. L. (1986). A history and theory of informed consent. Oxford University Press.

[bibr19-15562646221076764] Faden R. R. Kass N. E. Goodman S. N. Pronovost P. Tunis S. Beauchamp T. L. (2013). An ethics framework for a learning health care system: *A Departure from Traditional Research Ethics and Clinical Ethics*. Hastings Center Report, 43(s1), S16–S27. 10.1002/hast.134 23315888

[bibr20-15562646221076764] Faherty L. J. Lurie N. Wong C. A. (2020). The COVID-19 ‘return-to-learning’ natural experiment. JAMA Health Forum, 1(10), e201211. 10.1001/jamahealthforum.2020.1211 36218547

[bibr21-15562646221076764] Finkelstein J. A. Brickman A. L. Capron A. Ford D. E. Gombosev A. Greene S. M. Peter Iafrate R. Kolaczkowski L. Pallin S. C. Pletcher M. J. Staman K. L. Vazquez M. A. Sugarman J. (2015). Oversight on the borderline: quality improvement and pragmatic research. Clinical Trials, 12(5), 457–466. 10.1177/1740774515597682 26374685 PMC4699562

[bibr22-15562646221076764] Fischhoff B. (2020). Making decisions in a COVID-19 world. JAMA, 324(2), 139. 10.1001/jama.2020.10178 32496560

[bibr23-15562646221076764] Flory J. H. Mushlin A. I. Goodman Z. I. (2016). Proposals to conduct randomized controlled trials without informed consent: A narrative review. Journal of General Internal Medicine, 31(12), 1511–1518. 10.1007/s11606-016-3780-5 27384536 PMC5130947

[bibr24-15562646221076764] Gallo A. Weijer C. White A. Grimshaw J. M. Boruch R. Brehaut J. C. Donner A. Eccles M. P. McRae A. D. Saginur R. Zwarenstein M. Taljaard M. (2012). What Is the role and authority of gatekeepers in cluster randomized trials in health research? Trials, 13(1), 116. 10.1186/1745-6215-13-116 22834691 PMC3443001

[bibr25-15562646221076764] Gopichandran V. Luyckx V. A. Biller-Andorno N. Fairchild A. Singh J. Tran N. Saxena A. Launois P. Reis A. Maher D. Vahedi M. (2016). Developing the ethics of implementation research in health. Implementation Science: IS, 11(1), 161. 10.1186/s13012-016-0527-y 27938400 PMC5148832

[bibr26-15562646221076764] Hutton J. L. (2001). Are distinctive ethical principles required for cluster randomized controlled trials? Statistics in Medicine, 20(3), 473–488. 10.1002/1097-0258(20010215)20:3<473::AID-SIM805>3.0.CO;2-D 11180314

[bibr27-15562646221076764] Kilama W. L. (2010). Health research ethics in malaria vector trials in Africa. Malaria Journal, 9(3), S3. 10.1186/1475-2875-9-S3-S3 21144083 PMC3002141

[bibr28-15562646221076764] King G. Gakidou E. Imai K. Lakin J. Moore R. T. Nall C. Ravishankar N. Vargas M. Téllez-Rojo M. M. Ávila J. E. H. (2009). Public policy for the poor? A randomised assessment of the Mexican universal health insurance programme. The Lancet, 373(9673), 1447–1454. https://pubmed.ncbi.nlm.nih.gov/19359034/10.1016/S0140-6736(09)60239-7 19359034

[bibr29-15562646221076764] Langston E. (2016). Voters in This Florida County Just Approved GM Mosquitoes to Fight Zika.” *Mother Jones*. Retrieved April 11, 2018 (https://www.motherjones.com/environment/2016/11/key-haven-monroe-county-florida-gm-mosquitos-2016-election/).

[bibr30-15562646221076764] Lignou S. (2018). Informed consent in cluster randomised trials: New and common ethical challenges. Journal of Medical Ethics, 44(2), 114–120. 10.1136/medethics-2017-104249 28780528

[bibr31-15562646221076764] MacKay D. Chakrabarti A. (2019). Government policy experiments and informed consent. Public Health Ethics, 12(2), 188–201. 10.1093/phe/phy015

[bibr32-15562646221076764] Manning W. G. Newhouse J. P. Duan N. Keeler E. B. Leibowitz A. (1987). Health insurance and the demand for medical care: evidence from a randomized experiment. The American Economic Review, 77(3), 251–277. https://www.jstor.org/stable/1804094 10284091

[bibr33-15562646221076764] Manson N. C. O’Neill O. (2007). Rethinking informed consent in bioethics. Cambridge University Press.

[bibr34-15562646221076764] McMahon S. A. Winch P. J. (2018). Systematic debriefing after qualitative encounters: An essential analysis step in applied qualitative research. BMJ Global Health, 3(5), e000837. 10.1136/bmjgh-2018-000837 PMC6135453 30233833

[bibr35-15562646221076764] McRae A. D. Taljaard M. Weijer C. (2016). Cluster-Randomized trials: A closer Look. Clinical Trials: Journal of the Society for Clinical Trials, 13(3), 294–300. 10.1177/1740774516629405 26931364

[bibr36-15562646221076764] Merriam S. B. (1998). *Qualitative Research and Case Study Applications in Education. Revised and Expanded from “Case Study Research in Education.”* Jossey-Bass Publishers, 350 Sansome St, San Francisco, CA 94104.

[bibr37-15562646221076764] Miller F. Wertheimer A. (2010). The ethics of consent: theory and practice. Oxford University Press.

[bibr38-15562646221076764] National Commission for the Protection of Human Subjects of Biomedical and Behavioral Research (1979). *The Belmont Report* . https://www.hhs.gov/ohrp/regulations-and-policy/belmont-report/index.html

[bibr39-15562646221076764] Newhouse J. P. Normand S.-L. T. (2017). Health policy trials. New England Journal of Medicine, 376(22), 2160–2167. 10.1056/NEJMra1602774 28564562

[bibr40-15562646221076764] O’Neill O. (2003). Some limits of informed consent. Journal of Medical Ethics, 29(1), 4–7. 10.1136/jme.29.1.4 12569185 PMC1733683

[bibr41-15562646221076764] Onwujekwe O. Shu E. Okonkwo P. (1999). Can community Leaders’ preferences Be used to proxy those of the community as a whole? Journal of Health Services Research & Policy, 4(3), 133–138. 10.1177/135581969900400303 10538877

[bibr42-15562646221076764] Patton M. Q. (1990). Qualitative evaluation and research methods (2nd Ed). Sage Publications, Inc.

[bibr43-15562646221076764] Petticrew M. Cummins S. Ferrell C. Findlay A. Higgins C. Hoy C. Kearns A. Sparks L. (2005). Natural experiments: An underused tool for public health? Public Health, 119(9), 751–757. 10.1016/j.puhe.2004.11.008 15913681

[bibr44-15562646221076764] Powell-Jackson T. Hanson K. Whitty C. J. M. Ansah E. K. (2014). Who benefits from free healthcare? Evidence from a randomized experiment in Ghana. Journal of Development Economics, 107, 305–319. 10.1016/j.jdeveco.2013.11.010

[bibr45-15562646221076764] Pratt B. Paul A. Hyder A. A. Ali J. (2017). Ethics of health policy and systems research: A scoping review of the literature. Health Policy and Planning, 32(6), 890–910. 10.1093/heapol/czx003 28335031

[bibr46-15562646221076764] Schaber P. Müller A. (2018). The routledge handbook of the ethics of consent. Routledge.

[bibr47-15562646221076764] Starr P. (2020). Using controlled trials to resolve Key unknowns about policy during the COVID-19 pandemic. JAMA, 323(23), 2369. 10.1001/jama.2020.8573 32463427

[bibr48-15562646221076764] Taljaard M. Hemming K. Shah L. Giraudeau B. Grimshaw J. M. Weijer C. (2017). Inadequacy of ethical conduct and reporting of stepped wedge cluster randomized trials: results from a systematic review. Clinical Trials: Journal of the Society for Clinical Trials, 14(4), 333–341. 10.1177/1740774517703057 28393537

[bibr49-15562646221076764] Taljaard M. Weijer C. Grimshaw J. M. Eccles M. P. (2013). The Ottawa statement on the ethical design and conduct of cluster randomised trials: précis for researchers and research ethics committees. BMJ, 346, f2838. 10.1136/bmj.f2838 23661113

[bibr50-15562646221076764] Taylor H. A. Pronovost P. J. Faden R. R. Kass N. E. Sugarman J. (2010). The ethical review of health care quality improvement initiatives: findings from the field. Issue Brief (Commonw Fund), 95, 1–12. https://www.researchgate.net/profile/Nancy-Kass/publication/45796622_The_ethical_review_of_health_care_quality_improvement_initiatives_findings_from_the_field/links/0fcfd5093c64bc695d000000/The-ethical-review-of-health-care-quality-improvement-initiatives-findings-from-the-field.pdf 20726137

[bibr51-15562646221076764] U.S. Government (1949). *Nürnberg Code of Ethics - Trials of War Criminals before the Nuremberg Military Tribunals under Control Council LawNo. 10”, Vol. 2, Pp. 181-182*. Washington.

[bibr52-15562646221076764] Wanless D. (2004). Securing good health for the whole population. HM Stationery Office London.

[bibr53-15562646221076764] Weijer C. Emanuel E. J. (2000). Protecting communities in biomedical research. Science, 289(5482), 1142–1144. 10.1126/science.289.5482.1142 10970227

[bibr54-15562646221076764] Weijer C. Grimshaw J. M. Taljaard M. Binik A. Boruch R. Brehaut J. C. Donner A. Eccles M. P. Gallo A. McRae A. D. Saginur R. Zwarenstein M. (2011). Ethical issues posed by cluster randomized trials in health research. Trials, 12(1), 100. 10.1186/1745-6215-12-100 21507237 PMC3107798

[bibr55-15562646221076764] World Medical Association (1964). *Declaration of Helsinki - Ethical Principles for Medical Research Involving Human Subjects*. Helsinki.

